# Family resilience influences on individual physical activity, diet and sleep quality: Family health climate and biobehavioural reactivity as driving mediators

**DOI:** 10.1371/journal.pone.0322612

**Published:** 2025-05-05

**Authors:** Mary Su-Lynn Chew, Dhiya Mahirah, Yi-Ching Lynn Ho, Kinjal Doshi

**Affiliations:** 1 Centre for Population Health Research and Implementation, Singapore Health Services, Singapore, Singapore; 2 Department of Psychology, National University Singapore, Singapore, Singapore; Lorestan University, IRAN, ISLAMIC REPUBLIC OF

## Abstract

The family is a crucial contributor to mental health and physical well-being. While bidirectional relationships between physical activity, diet, sleep, and mental well-being are well-documented, the influence of the family on these behaviours is less explored. This study aimed to examine the relationships between family resilience and individual lifestyle behaviours in community-dwelling adults, focusing on the roles of a supportive family health climate and reduced biobehavioural reactivity. Data were collected from 200 family dyads in Singapore through questionnaires assessing family resilience, health climate related to physical activity and nutrition, individual lifestyle behaviours (physical activity, diet, sleep), and demographics. Structural equation modelling was used to explore the connections between family resilience and individual lifestyle behaviours, particularly how family health climate and biobehavioural reactivity mediate these relationships. Participants included 200 dyads with a mean age of 42 years (SD = 15.18; range 15–85), 62.7% female and 67.3% with tertiary education. 85.5% were Chinese households and 83% lived in public housing. Family resilience was associated with increased engagement in physical activity, diet quality, and sleep quality, with higher engagement linked to lower biobehavioural reactivity and a healthier family health climate. Significant mediation effects were observed, with family resilience positively influencing physical activity (total indirect effect: β = .164, p < .001) and diet quality (β = .113, p = .004) through family health climate, while negatively impacting sleep quality (β = -.168, p < .001) mediated by biobehavioural reactivity. This study highlights that family dynamics significantly influence individual physical activity, dietary habits, and sleep quality, thereby enhancing health outcomes. Specifically, family resilience promotes healthier lifestyle behaviours by fostering a supportive family health climate and reducing biobehavioural reactivity. Interventions aimed at strengthening family resilience may thus provide a cost-effective strategy for improving population health.

## Introduction

Families are units of individuals related by blood, marriage, or adoption living in the same household. Even though family members are independent, they influence each other and the family system [1]. This interconnectedness means that negative family influences, such as conflict, poor communication, and lack of support, can undermine individual well-being and contribute to mental health difficulties [2–4]. Such dynamics can lead to increased stress, anxiety, and unhealthy coping mechanisms, resulting in poorer health behaviours such as poor diet and lack of physical activity, and overall mental health outcomes [5,6]. The time spent among family members and the interwoven nature of family activities affects individual family members’ health behaviours and practices through a family systems approach. The family health climate (FHC), defined as the shared perceptions of health-related practices within the family, highlights how behaviours and interactions shape individual health-related behaviours like physical activity and dietary habits both within and outside the family [7].

Similarly, communication between family members during time shared through routines, rituals, and recreation are important factors for establishing and building family resilience and the family’s emotional health [8]. Family resilience is a protective factor and can be seen as the family’s capacity, as a functional system, to withstand adversity and emerge stronger as a result [9]. Components of family resilience like shared meaning-making, communication clarity, emotional sharing, and problem-solving are mutually interactive and synergistic. This allows flexibility and change as family members mobilise within their family system and adapt to emerging challenges within their social environment [10]. In contrast, families that lack positive components such as effective communication and emotional support may face conflicts, misunderstandings, and disconnection, which can undermine their resilience and overall well-being. Research indicates that individuals from families with a balance of cohesion, flexibility, and strong communication are more resilient, highlighting the critical role of supportive family dynamics [11].

The biobehavioural family model (BBFM) integrates the biopsychosocial perspective with the illness treatment by identifying specific pathways through which family relationships (i.e., family resilience and family health climate) impact disease activity via psychobiological mechanisms like depression and anxiety (i.e., biobehavioural reactivity) [12]. Building upon the understanding provided by the BBFM framework of how family-level factors like family resilience and family health climate can impact individual health outcomes through biobehavioural pathways, this study aims to expand on that framework by examining if family resilience can lead to better engagement in healthy lifestyle behaviours like physical activity, diet, and sleep through the mediating roles of family health climate and biobehavioural reactivity. This is motivated by prior studies that have found bidirectional relationships between mental health and these specific health behaviours [13]. For instance, research has shown that engaging in regular exercise can help prevent and manage symptoms of depression and anxiety [14], while conversely, poor mental health has been linked to less healthy dietary habits and poorer sleep quality [15,16]. Poor sleep quality may also heighten the likelihood of anxiety disorders and depression [17].

Despite several studies examining the associations between negative family functioning and mental health difficulties with poorer lifestyle behaviours and disease outcomes, there is limited evidence in translational research on how positive family influences can serve as protective factors [18]. The current study aimed to close this gap by analysing the influence of family resilience on individual physical activity, diet, and sleep through the mediating role of biobehavioural reactivity and family health climate. We hypothesised that higher levels of family resilience would result in greater physical activity, better diet quality, and improved sleep quality. Each of these outcomes will be tested through separate mediation models that examine the pathways involving a healthier family climate and reduced biobehavioural reactivity. This is motivated by the established bi-directional relationship between mental and physical well-being, suggesting that leveraging family mechanisms may lead to better engagement and sustained healthy lifestyle changes compared to individual-focused interventions [12].

Understanding and identifying the mechanisms through which family resilience and its components contribute to healthier behaviours will help us create effective interventions that enhance family dynamics with the end goal of improving health outcomes.

## Methods

### Participants & procedures

This study is based on data collected from a cross-sectional survey conducted in Singapore by a commercial survey vendor from 26 October 2020 to 15 January 2021 during the Coronavirus Disease 2019 (COVID-19) pandemic and has been used in earlier studies [19,20]. The study recruited 200 family dyads (i.e., 400 individuals) living in the same household to participate in an online survey conducted in English, consisting of self-reported measures and demographic characteristics of the participants. Due to the COVID-19 pandemic restrictions, recruitment and data collection by the vendor was conducted using remote measures only, including the use of Facebook advertisements, the survey vendor’s participant email database, and a secure online survey platform. We conducted eligibility checks to ensure that we recruited only family dyads that met the following inclusion criteria: they were Singaporean citizens or permanent residents aged 15 years and above who were both living in the same household. Written informed consent from participants and participants’ legal guardians/next of kin was not required to participate in this study in accordance with the national legislation and the institutional requirements. Quality checks and the discarding of erroneous responses were also performed before the de-identified dataset was sent to the study team. The study was approved by the institutional ethics committee (CIRB Ref. 2020/2195). A minimum sample size of 279 individuals is required for parallel mediation analyses of 2 mediators and 16 measured variables for 80% power and a 95% confidence interval, assuming a moderate effect size and variances of 1 for the outcome variable and both mediators [21,22].

### Measures

Participants provided data on their demographic characteristics (sex, age, education level, household race, number of people living in the household, type of housing (i.e., public or private housing), and household income) and completed the following self-reported measures.

#### Family Resilience Assessment Score (FRAS).

The FRAS is a 54-item validated questionnaire that measures distinct family processes based on Walsh’s (2006) Family Resilience Framework [23,24]. The instrument has been validated in Singapore with good internal consistency (*α *= 0.92) and construct validity [25], yielding a 7-factor model that accounted for 83% of variance with the following factors: (1) meaning-making and positive outlook (MMPO), (2) transcendence and spirituality (TS), (3) flexibility and connectedness (FLCO), (4) resources-community (R-C), (5) resources-neighbours (R-N), (6) clarity and open emotional expression (COEE), and (7) collaborative problem-solving (CPS). The items are rated on a 4-point Likert scale (1 = “Strongly disagree” to 4 = “Strongly agree”). Higher scores on the questionnaire indicate higher levels of family resilience.

#### Family health climate - Singapore (FHC (Sg)).

The FHC (Sg) scales [19] is an adapted 33-item questionnaire from the original FHC scales [26] that measures both the physical activity (3-factor model accounting for 71.2% variance) and nutrition (4-factor model accounting for 72.8% variance) domain of a family’s health climate, including subdomains like value, communication, cohesion, information, and consensus. The scales are validated in Singapore with good item discriminant validity, internal consistency reliability (*α *> 0.7 threshold), construct validity, and measurement invariance [19]. The questionnaire used a four-point Likert scale rating of 0 = “strongly disagree”, 1 = “somewhat disagree”, 2 = “somewhat agree”, 3 = “strongly agree”. The range of possible scores for FHC (Sg) was from 0 to 99, with higher scores indicating a better family health climate.

#### Patient Health Questionnaire (PHQ-ADS).

The PHQ-ADS questionnaire [27] comprises the well-validated PHQ-9 [28] and GAD-7 [29] self-administered screening tools, which contain 9 and 7 items respectively for depressive-anxiety symptomology in the previous two weeks. The PHQ-ADS complements the individual PHQ-9 and GAD-7 scales by providing a measure of overall psychological symptomology for an individual [27]. The questionnaire is scored on a 4-point Likert scale (0 = not at all, and 3 = nearly every day), with scores ranging from 0 to 48. Higher scores reflect more depressive and anxiety symptoms, with cut-off scores of 10, 20, and 30 indicating mild, moderate, or severe levels of depressive-anxiety symptomology. The use of PHQ-ADS has good internal reliability (*α* = 0.8 to 0.9) and strong convergent and construct validity for use in studies in which a single depressive/anxiety summative score is desirable [27].

#### International Physical Activity Questionnaire (IPAQ).

The IPAQ consists of 27 items that assess the individual time spent on physical activity in the past week. It has shown acceptable test-retest reliability and criterion validity across countries [30]. We used the IPAQ scoring protocol to calculate physical activity-related energy expenditures (Metabolic Equivalent of Task (MET)-minutes/week), utilising the recreation and leisure domain score. Higher MET-minutes/week indicate more physical activity in minutes per week.

#### Diet Screener.

The 37-item diet screener [31] assesses individual consumption of selected food groups in the past year. Food items included come from seven major food groups: grains, protein, dairy, fruits, vegetables, sugary or fatty foods, and sweetened beverages. The diet screener showed reasonably good validity and good reproducibility when compared with the 163-item Food Frequency Questionnaire in Singapore residents [31].

We calculated the total score using the Dietary Approaches to Stop Hypertension (DASH) scoring index [32] that accounted for key food groups, with a score of 1–5 for each group: high intake of whole grains, fruits, vegetables, nuts, legumes, low-fat dairy, and low intake of red and processed meats (reversed), and sweet beverages (reversed scoring). Sodium intake was excluded from the total DASH score, as the diet screener may not reliably assess it [31]. DASH scores range from 7 to 35, with higher scores indicating a higher consumption frequency of healthy food and a lower consumption frequency of unhealthy food compared to other participants.

#### Pittsburgh Sleep Quality Index (PSQI).

The PSQI [33] is a 19-item questionnaire to assess sleep quality and sleep disturbance for one month. It consists of seven components: subjective sleep quality, sleep latency, sleep duration, habitual sleep efficiency, sleep disturbances, use of sleeping medication, and daytime dysfunction. The questionnaire has shown acceptable internal homogeneity, test-retest reliability, and validity when tested in a sample of healthy and non-healthy patients over an 18-month period [33]. PSQI scores range from 0 to 21, with a higher score indicating poorer sleep quality.

### Data analysis

Data was analysed using STATA version 17.0 (STATA Corp LLC, TX, USA). Descriptive statistics and Pearson’s correlation tests were conducted to determine the characteristics of the measured variables. We built a Structural Equation Modelling (SEM) framework using maximum likelihood to examine the relationship between family resilience and respective individual lifestyle behaviours (i.e., physical activity, diet, and sleep quality).

**Fig 1:**
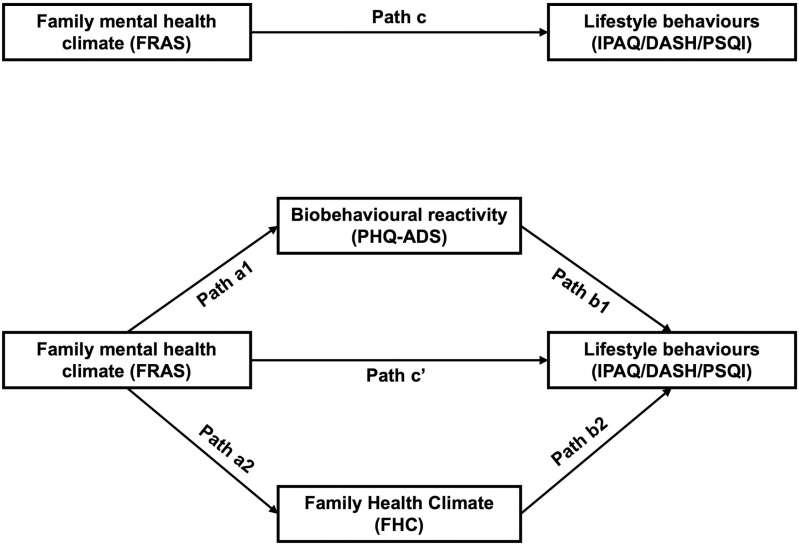
Conceptual model of parallel mediation analyses.

Two parallel mediation pathways were fitted to determine the influence of an individual’s biobehavioural reactivity and family health climate on this link. In our conceptual model (Fig 1), the observed effect of family emotional health climate (i.e., Family Resilience Assessment Scale (FRAS)) on lifestyle behaviours is called the total effect (path c). The total effects comprised of a direct effect pathway (path c’) of family emotional health climate on lifestyle behaviours and a total indirect effects pathway (mediated: path a1b1 + path a2b2) of family emotional health climate on lifestyle behaviours through family health climate and biobehavioural reactivity.

Erroneous data entry for individual sleep quality was treated as missing data (n = 6) and used maximum likelihood with missing values for the models using this outcome variable. To address the discrepancy in household income (n = 36 dyads) and housing type (n = 3 dyads) among family dyad responses, we cleaned the data to take the older and male participants’ responses as tiebreakers.

Skew and kurtosis were assessed with the recommended cut-off ranges of -3 to + 3 and -10 to + 10 of the variables to determine if the data distribution was not severely non-normal [34]. Covariates were identified according to the individual and family level variables (i.e., X, M, Y); age, sex, education level, household race, number of people living in the household, and housing type were included in the models. The reference groups for the categorical covariates were females, Chinese household race, 3–4 room public housing flats, and tertiary education.

Simple mediation models with covariates were also built to determine the specific indirect effect of each mediator. To examine the parallel and simple mediation effects, we implemented 95% confidence intervals for the bootstrapped estimates conducted with 5,000 resamples [35] for the total indirect effect of both mediators and the indirect effect of each mediator. Post-hoc analyses were conducted to identify which of the FRAS seven subdomains had the strongest influence on the outcome variable. Separate parallel mediation models were fitted for each subdomain, assessing standardised coefficients for the pathways to the mediators and from the mediators to the outcome.

Model fit was assessed using criteria such as chi-square (χ^2^) over degrees of freedom (df) values less than or equal to 2, comparative fit index (CFI) values greater than or equal to 0.95, Tucker-Lewis index (TLI) values greater than or equal to 0.90, root mean square error approximation (RMSEA) values less than or equal to 0.06, and standardised root mean square residual (SRMR) values less than or equal to 0.08) were used to determine a reasonably good model fit [36].

Finally, considering the existing literature, we tested for bidirectional pathways by inverting our original model pathways (i.e., swapped X ◊ Y) to determine if significant mediations existed in the other direction (See S1 Appendix for summary results on the parallel mediation models with inversed pathways).

## Results

A total of 200 dyads living in the same household responses were collected. The mean age of participants was 42 years (SD = 15.18; range 15–85 years), 62.7% were female, and 67.3% had tertiary education. Females and those with tertiary education in the sample were primarily overrepresented compared to the local population (51.1% females and 32.4% tertiary education) [37].

The between-dyad relationships of participants living in the same household comprised of parent-child (43%), couples (37.5%), siblings (19%), and aunt-nephew (0.5%). 85.5% came from Chinese households, 5.5% from Indian households, 5% from Malay households, and 4% from Mixed Race or Other households. 1% lived in 1 or 2-room public housing flats, 50% lived in 3 or 4-room public housing flats, 32% lived in 5-room or executive public housing flats, and 17% lived in private housing. 10.5% had household incomes ranging from $0 to $2,500, 40.5% from $2,500 to $7,500, and the rest (49%) had above $7,500 (median household income in 2020 was $7,744 [38]). Of those who reported household incomes ranging from $0 to $2,500, all lived in public housing, and most (81%) lived in 3–4-room public housing flats.

### Descriptive statistics and correlations of variables

Table 1 provides information about the measured variables, including mean, standard deviation, and minimum and maximum scores. Skew and kurtosis of the measured variables fall within Kline’s recommended cut-off ranges [34].

**Table 1:** Descriptive statistics of the measured variables.

Variable	Mean	SD	Min	Max	Skew	Kurtosis
Family resilience	167.52	19.82	112	216	0.12	2.61
Family health climate	61.71	15.50	7	96	-0.26	3.21
Biobehavioural reactivity	9.49	9.81	0	48	1.39	4.83
Individual physical activity	203.69	159.00	0	540	0.55	2.61
Individual diet quality	20.80	4.89	9	34	0.11	2.53
Individual sleep quality	5.71	3.71	0	19	0.88	3.26

Note: Figures are rounded to 2 decimal places.

Table 2 presents the relationship between the measured variables. Family resilience and family health climate were positively correlated with individual lifestyle behaviours and negatively correlated with biobehavioural reactivity. All correlation coefficients were below 0.90, suggesting no multicollinearity among these variables for path analyses.

**Table 2:** Correlation between family resilience, family health climate, biobehavioural reactivity and individual lifestyle behaviours.

	1.	2.	3.	3a.	3b.	4.	5.	6.
1. Family resilience	1							
2. Family health climate	0.57**	1						
3. Biobehavioural reactivity	-0.26**	-0.20**	1					
3a. Depressive symptoms	-0.25**	-0.20**	–	1				
3b. Anxiety symptoms	-0.24**	-0.18**	–	0.77**	1			
4. Individual physical activity	0.16**	0.28**	-0.03	-0.06	0.001	1		
5. Individual diet quality	0.21**	0.25**	-0.12*	-0.15**	-0.07	0.10*	1	
6. Individual sleep quality	-0.16**	-0.15**	0.58**	0.58**	0.51**	-0.01	-0.16*	1

Notes: *p < 0.05; **p < 0.01. Figures are rounded to 2 decimal places.

### Parallel mediation models of respective lifestyle behaviours and covariates

The original models highlight the proactive role of family resilience in influencing health behaviours (Table 3), while the inversed models reveal the impact of health climate and behaviours on family resilience (S1 Appendix). Although the inversed models identified a new unidirectional mediation pathway, they exhibited poorer overall fit compared to the original models (S1 Appendix).

**Table 3:** Mediation effects of PHQ-ADS and FHC with lifestyle behaviours.

Model	Path	β	SE B	p-value
Family resilience to individual physical activity (X → M1|M2 → Y1)	Total effect (c)	.157	.38	**.001**
	Direct effect (c’)	-.0074	.50	.91
	Total indirect effect (a x b)	.164	.34	**<.001**
(X → M1 → Y1)	M1 indirect effect	.0098	.11	.48
(X → M2 → Y1)	M2 indirect effect	.161	.33	**<.001**
Family resilience to individual diet quality (X → M1|M2 → Y2)	Total effect (c)	.209	.012	**<.001**
	Direct effect (c’)	.096	.015	.15
	Total indirect effect (a x b)	.113	.0010	**.004**
(X → M1 → Y2)	M1 indirect effect	.0089	.0037	.48
(X → M2 → Y2)	M2 indirect effect	.109	.0097	**.005**
Family resilience to individual sleep quality (X → M1|M2 → Y3)	Total effect (c)	-.149	.0090	**.002**
	Direct effect (c’)	.019	.010	.72
	Total indirect effect (a x b)	-.168	.0081	**<.001**
(X → M1 → Y3)	M1 indirect effect	-.152	.0058	**<.001**
(X → M2 → Y3)	M2 indirect effect	-.046	.0065	.14

Notes: X = family resilience; M1 = PHQ-ADS; M2 = FHC; Y = lifestyle behaviours; β = standardised regression coefficient; SE B = bootstrap standard error; M1|M2  = M1 and M2 as parallel mediators.

Table 3 presents the mediating effects of biobehavioural reactivity and family health climate on individual physical activity, diet quality, and sleep quality, respectively. Table 4 shows the overall goodness-of-fit indices for the respective parallel mediation models.

**Table 4:** Goodness-of-fit model indices for parallel mediation models.

Model	χ^2^/df	CFI	TLI	RMSEA	SRMR	Overall model fit
Family resilience to individual physical activity (X → M1|M2 → Y1)	27.17/25	0.993	0.985	0.015 (*p *> .05)	0.023	Good
Family resilience to individual diet quality (X → M1|M2 → Y2)	27.72/25	0.991	0.981	0.017 (*p* > .05)	0.024	Good
Family resilience to individual sleep quality (X → M1|M2 → Y3)	40.88/25	0.964	0.923	0.040 (*p* > .05)	–	Good

Notes: X = family resilience; M1 = PHQ-ADS; M2 = FHC; Y = lifestyle behaviours; M1|M2  = M1 and M2 as parallel mediators.

Regarding the covariates across the models for the family resilience, family health climate and biobehavioural reactivity variables, participants from Indian and Malay households had significantly higher family resilience scores reported than participants from Chinese households (β = .190, *p* < .001, CI = .095,.286; β = .203, *p* < .001), and those living in private property (β = .192, *p* < .001, CI = .0996,.285), 5-room public housing flats (β = .129, *p* = .031, CI = .0118,.247) and 1–2 room public housing flats (β = .0878, *p* = .002, CI = .0324,.143) also had significantly higher family resilience scores than those living in 3–4 room public housing flats. Participants from Indian households had significantly lower family health climate scores than their Chinese counterparts (β = -.110, *p* = .02, CI = -.202, -.0176), while those living in 1–2 room public housing flats (β = .114, *p* < .001, CI = .0697,.158) had significantly higher family health climate scores than those living in 3–4 room public housing flats. The younger participants were, the higher biobehavioural reactivity scores were reported (β = -.281, *p* < .001, CI = -.367, -.195).

### Mediating effects of biobehavioural reactivity and family health climate on physical activity

**Fig 2:**
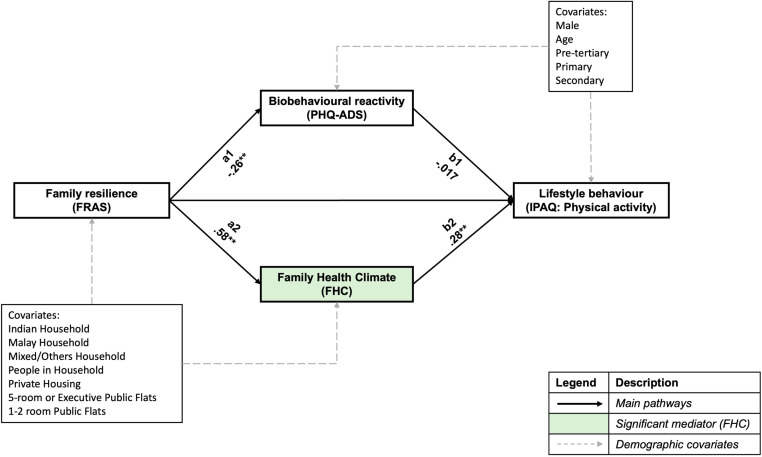
Parallel mediation model of family resilience with individual physical activity.

Fig 2 shows standardised path coefficients and their significance for the parallel mediation of family resilience, individual physical activity, and covariates. The overall model fit indices reported indicate a good model fit (Table 4). Significant mediation was found between family resilience and individual physical activity after controlling for covariates, with a significant total indirect effect of both mediators (β = .164, *p* < .001).

Further simple mediation analyses show that the family health climate is the driving mediator of the relationship between family resilience and individual physical activity levels, with a significant indirect effect found after bootstrapping (β = .161, *p* < .001). For one standard deviation change in family resilience, individual physical activity in leisure time increased by 0.161 standard deviation through the mediating effect of the family health climate. Younger participants reported higher individual physical activity (β = -.128, *p* = .012, CI = -.227, -.0284), while no other significant differences were observed for sex and education levels.

For post-hoc analysis, the indirect effects standardised coefficients and p-values from each family resilience subscale to individual physical activity through family health climate were as follows: 1) MMPO (β = .141, *p* < .001), 2) TS (β = .0648, *p* = .001), 3) FLCO (β = .127, *p* < .001), 4) R-C (β = .101, *p* < .001), 5) R-N (β = .0769, *p* < .001), 6) COEE (β = .154, *p *< .001) and 7) CPS (β = .161, *p* < .001).

### Mediating effects of biobehavioural reactivity and family health climate on diet quality

**Fig 3:**
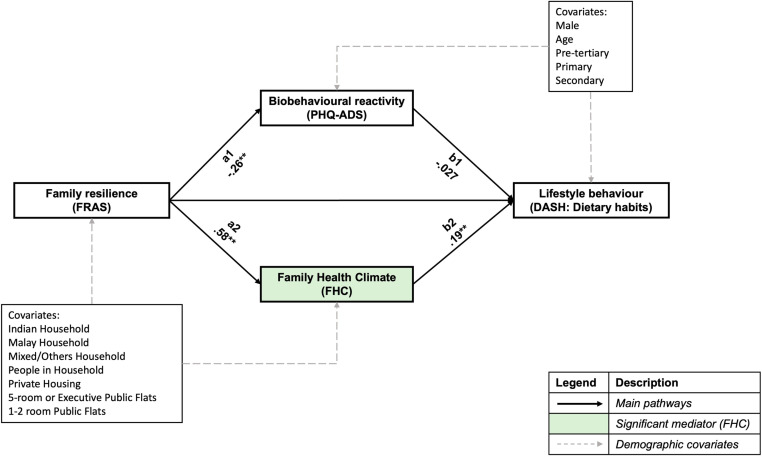
Parallel mediation model of family resilience with individual diet quality.

Fig 3 shows standardised path coefficients and their significance for the parallel mediation of family resilience and individual diet quality and covariates. The overall model of fit indices reported indicates a good model fit (Table 4). Significant mediation was found between family resilience and diet quality after controlling for covariates, with a significant total indirect effect of both mediators (β = .113, *p* = .004).

Further simple mediation analyses show that the family health climate is the driving mediator of the relationship between family resilience and individual diet quality, with a significant indirect effect found after bootstrapping (β = .109, *p* = .005). For one standard deviation change in family resilience, individual diet quality increased by 0.109 standard deviation through the mediating effect of the family health climate.

In terms of individual diet quality, males reported significantly lower DASH scores than females (β = -.106, *p* = .023, CI = -.198, -.0148), and those with primary education also reported significantly lower DASH scores than those with tertiary education (β = -.119, *p* = .01, CI = -.210, -.0285). Conversely, among older participants, higher DASH scores were reported (β = .177, *p* < .001, CI = .0789,.274).

For post-hoc analysis, the indirect effects standardised coefficients and p-values from each family resilience subscale to individual diet quality through family health climate were as follows: 1) MMPO (β = .111, *p *< .001), 2) TS (β = .0546, *p* = .002), 3) FLCO (β = .0986, *p* < .001), 4) R-C (β = .0807, *p* < .001), 5) R-N (β = .0732, *p* < .001), 6) COEE (β = .105, *p* = .002) and 7) CPS (β = .109, *p* = .001).

### Mediating effects of biobehavioural reactivity and family health climate on sleep quality

**Fig 4:**
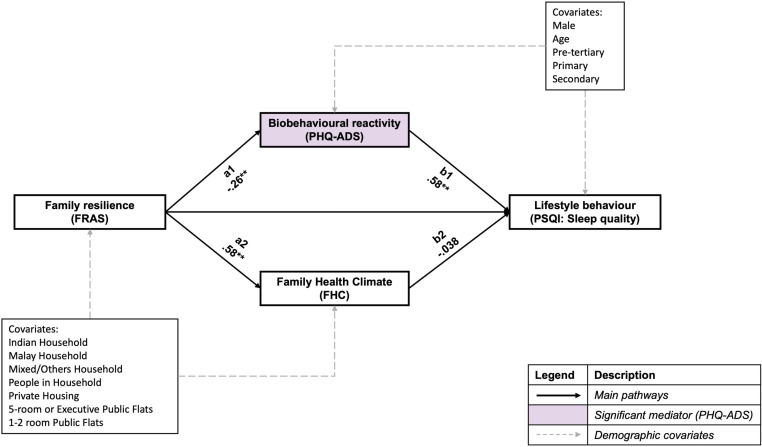
Parallel mediation model of family resilience with individual sleep quality.

Fig 4 shows standardised path coefficients and their significance for the parallel mediation of family resilience and individual sleep quality and covariates. The overall model of fit indices reported indicates a good model fit (Table 4). Significant mediation was found between family resilience and sleep quality after controlling for covariates, with a significant total indirect effect of both mediators (β = -.168, *p* < .001).

Further analyses show that biobehavioural reactivity is the driving mediator of the relationship between family resilience and individual sleep quality, with a significant specific indirect effect found after bootstrapping (β = -.152, *p* < .001). For one standard deviation change in family resilience, individual sleep quality (i.e., higher PSQI scores indicate poorer sleep quality) improved by 0.152 standard deviation through the mediating effect of biobehavioural reactivity. Regarding individual sleep quality, no sex, age, or education differences were observed.

For post-hoc analysis, the indirect effects standardised coefficients and p-values from each family resilience subscale to individual sleep quality through biobehavioural reactivity were as follows: 1) MMPO (β = -.174, *p *< .001), 2) TS (β = .0149, *p* > .05), 3) FLCO (β = -.164, *p* < .001), 4) R-C (β = -.128, *p* < .001), 5) R-N (β = -.0395, *p* > .05), 6) COEE (β = -.152, *p* < .001) and 7) CPS (β = -.141, *p* < .001).

## Discussion

The present study took an integrative approach to evaluate how family health climate and individual biobehavioural reactivity mediate the relationship between family resilience and multiple health behaviours. Specifically, we tested three distinct mediation models to better understand complex family dynamics. Our findings revealed that higher family resilience was associated with increased engagement in physical activity and diet quality. These relationships were mediated by the family health climate, suggesting that a positive and supportive family environment regarding health plays a key role in translating family resilience into healthier behaviours like more physical activity and better diet quality.

In contrast, higher family resilience led to better sleep quality through a different pathway, with lower biobehavioural reactivity acting as the mediator. Members of resilient families tend to develop more adaptive emotion regulation and coping skills, which enhance their stress management and result in healthier sleep patterns [39–41]. These findings align with the BBFM framework, which posits that managing biobehavioural reactivity and cultivating a positive family health climate is crucial for amplifying the positive effects of family resilience on individual health behaviours [12].

### Role of demographic factors in family resilience and health behaviours

Demographic differences highlight the influence of cultural and socioeconomic factors on family resilience and health behaviours. The nonlinear relationship between housing type (a proxy for socioeconomic status; [42]) and family resilience indicates that beyond income, cultural resources and coping strategies play a role. Notably, both lower and higher socioeconomic groups reported greater resilience compared to middle-income households. Participants from Indian and Malay families exhibited higher resilience and lower family health climate scores than those from Chinese families, emphasising the impact of cultural values and family structures.

Variations at the individual level, such as increased physical activity among younger participants and differences in dietary habits based on age, gender, and education, further illustrate how demographic factors shape health behaviours. These findings can guide tailored interventions that consider cultural, socioeconomic, and developmental aspects. Importantly, these demographic factors were controlled for in the SEM, enabling a clearer analysis of the primary relationships.

### The influence of family resilience on family health climate and individual health behaviours

Our mediation findings underscore the significant role of family resilience in influencing individual physical activity levels and dietary behaviours through the family health climate, aligning with existing literature [43–45]. While previous studies acknowledge the multifactorial nature of healthy lifestyle behaviours and explore various potential driving factors, they often neglect to examine these relationships through a mediation model. Higher family resilience is positively linked to increased leisure time, greater physical activity, and improved dietary habits, as families high in resilience engage in routines that promote healthy lifestyles rooted in shared values and cultural norms [43]. Conversely, lower family resilience is associated with unhealthy behaviours, such as infrequent shared meals and less constructive communication, which undermine family health [45,46]

In contrast, our research explicitly examines how family resilience contributes to lifestyle behaviours through a healthier family health climate, providing clear pathways that enhance understanding of these protective mechanisms. The primary analyses indicate that family resilience significantly predicts individual physical activity and dietary habits. Additionally, post-hoc analyses revealed that specific family resilience components, such as collaborative problem solving, clarity and open emotional expression, meaning-making and positive outlook, and flexibility and connectedness, are important driving factors influencing these lifestyle choices, showing a strong positive impact on individual physical activity and dietary habits. These findings emphasise the importance of considering collective family attitudes and behaviours in shaping individual health habits. It is plausible that families engaging in collaborative problem-solving are better equipped to tackle health-related issues together. By working as a team to develop strategies, they exemplify flexibility and connectedness, creating a supportive environment that encourages regular physical activity and healthier lifestyle choices, ultimately leading to improved health outcomes. Similarly, families that prioritise open emotional expression can foster an atmosphere where members feel comfortable sharing challenges related to healthy eating and exercise. This acceptance of their feelings may enhance their willingness to persist in adopting healthier behaviours, as they find meaning in their experiences and maintain a positive outlook toward change. Ultimately, this supportive environment can lead to improved lifestyle choices and a stronger commitment to health.

Overall, family resilience serves as a protective factor, reinforcing the need for family-centred approaches in public health interventions [43]. While individual-level interventions have had mixed results, family-centred approaches addressing family-level influences like family health climate show greater effectiveness and sustainability in promoting physical activity and healthy nutrition [47–51]. Strengthening essential components of family resilience, like collaborative problem-solving, clarity in communication, and meaning-making, can effectively encourage healthy lifestyle choices and enhance individual levels of physical activity and dietary habits among families.

### Family resilience as a protective factor of emotional regulation and sleep quality

The influence of family resilience extends beyond physical health behaviours to emotional well-being and sleep quality. Research consistently links family resilience to mood, anxiety, and sleep quality. Families with low resilience report more emotional problems and poorer sleep, particularly during stressful periods such as crises or the COVID-19 pandemic [52–55]. Conversely, families with higher resilience tend to experience better psychological and sleep outcomes. Stressors like inadequate family functioning can contribute to parental distress, negatively affecting other family members, including children, and resulting in poorer sleep quality [56]. Emotional disturbances often manifest as sleep issues, including difficulties falling or staying asleep, and having fragmented sleep, resulting in inadequate rest [16].

Post-hoc analyses identified significant family resilience subscales influencing sleep quality through biobehavioural reactivity. Key contributors included meaning-making and positive outlook, flexibility and connectedness, and clarity and open emotional expression. Families that foster a positive outlook and strong emotional expression create a supportive environment essential for better sleep. Additionally, maintaining flexibility in family dynamics enhances adaptability to stressors, further promoting improved sleep quality. However, the non-significance of the transcendence and spirituality subscale, despite the overall significance of family resilience, suggests that this aspect may not independently contribute to resilience in this context or may be overshadowed by other, more influential factors, such as emotional support and effective communication within the family.

Our findings extend previous research by demonstrating that family resilience indirectly affects sleep quality, mediated through biobehavioural reactivity. While previous studies have established an association between psychological resilience and sleep quality [57], this study highlights a directional pathway: resilience influences sleep through its impact on biobehavioural reactivity. This pathway is further supported by research showing that higher family resilience correlates with better sleep quality and reduced depression in parents of children with epilepsy [40]. Overall, enhancing components of family resilience, such as emotional expression and flexibility, may be an effective strategy for mitigating sleep disturbances linked to emotional and psychological factors.

### Strengths and Limitations

The strengths of this study include its holistic approach to exploring overall family mechanisms, regardless of member roles, which was supported by prior evidence showing high concordance between family members’ scores on family health climate measures [19]. Individual scores effectively represented the family’s collective attitudes and behaviours, justifying the examination of the family. Further strengthening the validity of this family-level approach, the findings examining parent-child, couple, and sibling interactions separately yielded similar results in promoting healthy lifestyle behaviours [58–61]. The study’s comprehensive model examination, which looked at all hypothesised pathways, including inverse ones, found that the hypothesised direction formed a better overall model fit.

However, the study’s limitations include its cross-sectional design, which restricts the ability to make causal inferences, as well as the small standardised indirect effect sizes (< 0.3) in the significant mediation models, warranting cautious interpretation [62]. Lastly, the generalizability of the findings may be limited due to the specific context of the COVID-19 pandemic, the online survey administration, and the cross-sectional nature of the data, which may only partially represent the broader population or capture changes over time.

### Implications

This study contributes to the existing literature by elucidating how family dynamics affect individual psychobiological states and lifestyle behaviours [39,44,56,63]. Our findings highlight family resilience as a protective factor that enhances family health climate and biobehavioural reactivity, leading to healthier behaviours such as increased physical activity, improved nutrition, and better sleep quality. To benefit from these positive health outcomes, public health interventions should adopt a family-centred approach.

Community programmes could be developed to increase family resilience through effective communication, shared decision making, and emotional support, all key components essential for fostering environments conducive to active participation in physical activities and healthy eating. Such programmes could facilitate family bonding activities that provide opportunities for families to communicate effectively about setting healthy goals, planning nutritious meals together, and engaging in physical activities to support stress management. Additionally, community-based interventions that promote family bonding can effectively mitigate the effects of stress on sleep quality by encouraging families to establish regular bedtime routines, participate in calming activities together like mindfulness exercises, and create a supportive environment that promotes rest and relaxation.

Implementing preventive family-based interventions that consider environmental modifications and integrates strategies across multiple levels, including the individual, family, community and societal/policy systems, can comprehensively address the interlinked modifiable lifestyle factors of diet, sleep, and physical activity, given the limitations of direct interventions targeting single behaviours [64]. For instance, the Families Overcoming Under Stress (FOCUS) Family Resilience programme has been widely implemented across community health settings, healthcare, and schools, demonstrating effectiveness in reducing parental distress and improving family functioning over time [65–69]. The FOCUS programme effectively teaches key family resilience components from the FRAS, including meaning making and positive outlook, clarity and open emotional expression, and collaborative problem solving, which collectively enhance family functioning by fostering open communication, shared understanding, and adaptive coping strategies [66].

Future studies could explore additional family resilience components, such as flexibility and connectedness, which are important for promoting healthier lifestyle behaviours. Ultimately, evaluating whether such programmes lead to improved health states and behaviours will be essential, reinforcing the need for public health initiatives to prioritise family resilience as a critical component in enhancing overall well-being, particularly in promoting physical activity, nutrition, and sleep quality.

### Conclusion

Our study tested bi-directional models and found that family influences on individual biobehavioural reactivity, physical activity, diet, and sleep quality demonstrated a superior fit. These findings underscore the urgent need for holistic family-based interventions targeting factors like family resilience and health climate, emphasizing the need for culturally tailored approaches to enhance physical activity and diet quality [49].

We established that family resilience, encompassing key components such as meaning-making and positive outlook, clarity and open emotional expression, collaborative problem-solving, and flexibility and connectedness, plays a crucial role in influencing individual health outcomes. In our models, family health climate serves as a driving mediator for physical activity and nutrition, while biobehavioural reactivity mediates the relationship with sleep quality. Fostering these resilience components can promote healthier lifestyle choices, enhancing physical activity and nutrition, and creating environments that support adaptive responses to improve sleep quality.

To translate these insights into practice, it is essential to encourage the development of community programmes that enhance family resilience through effective communication, shared decision making, and emotional support. These programmes should also promote family bonding activities that encourage setting health goals, planning nutritious meals, engaging in physical activities, and establishing healthy sleep routines together.

Future research should explore diverse family profiles within the health climate context to tailor interventions effectively, particularly for families with low family health climate scores in physical activity or nutrition. Understanding the interplay of family-level factors, such as resilience and health climate, with individual-level factors like biobehavioural reactivity is essential for developing impactful, cost-effective public health strategies that promote family health.

## Supporting information

S1 Appendix Summary results of inversed parallel mediation models. This appendix contains the summary results of the inversed parallel mediation models in comparison with the study findings.
(DOCX)

## References

[1] Cox MJ, Paley B. Understanding families as systems. Curr Dir Psychol Sci. 2003 Oct 24;12(5):193–6.

[2] Sander JB, McCarty CA. Youth depression in the family context: familial risk factors and models of treatment. Clin Child Fam Psychol Rev. 2005;8(3):203–19. doi: 10.1007/s10567-005-6666-3 16151618 PMC1352328

[3] Yu Y, Yang X, Yang Y, Chen L, Qiu X, Qiao Z, et al. The role of family environment in depressive symptoms among university students: a large sample survey in China. PLoS One. 2015;10(12):e0143612. doi: 10.1371/journal.pone.0143612 26629694 PMC4667844

[4] Dmitrieva J, Chen C, Greenberger E, Gil‐Rivas V. Family relationships and adolescent psychosocial outcomes: converging findings from eastern and western cultures. J Res Adolesc. 2004 Dec 29;14(4):425–47.

[5] Sallis JF, Nader PR. Family determinants of health behaviors. In: Health Behavior. Boston, MA: Springer US; 1988. p. 107–24.

[6] Umberson D, Crosnoe R, Reczek C. Social relationships and health behavior across the life course. Annu Rev Sociol. 2010 Jun 1;36(1):139–57. 21921974 10.1146/annurev-soc-070308-120011 PMC3171805

[7] Wäsche H, Niermann C, Bezold J, Woll A. Family health climate: a qualitative exploration of everyday family life and health. BMC Public Health. 2021;21(1):1261. doi: 10.1186/s12889-021-11297-4 34187447 PMC8240432

[8] Black K, Lobo M. A conceptual review of family resilience factors. J Fam Nurs. 2008;14(1):33–55. doi: 10.1177/1074840707312237 18281642

[9] Walsh F. The concept of family resilience: crisis and challenge. Fam Process. 1996 Sep;35(3). 10.1111/j.1545-5300.1996.00261.x 9111709

[10] Walsh F. Family resilience: a developmental systems framework. Eur J Dev Psychol. 2016;13(3):313–24. doi: 10.1080/17405629.2016.1154035

[11] Carr K, Kellas JK. The role of family and marital communication in developing resilience to family-of-origin adversity. J Fam Commun. 2018 Jan 2;18(1):68–84.

[12] Wood BL, Woods SB, Sengupta S, Nair T. The biobehavioral family model: an evidence-based approach to biopsychosocial research, residency training, and patient care. Front Psychiatry. 2021 Oct 5;12. 10.3389/fpsyt.2021.725045 PMC8523802 34675826

[13] Hepsomali P, Groeger JA. Diet, sleep, and mental health: insights from the UK Biobank Study. Nutrients. 2021 Jul 27;13(8):2573. 34444731 10.3390/nu13082573 PMC8398967

[14] Mason OJ, Holt R. Mental health and physical activity interventions: a review of the qualitative literature. J Ment Health. 2012;21(3):274–84. doi: 10.3109/09638237.2011.648344 22533784

[15] O’Neil A, Quirk SE, Housden S, Brennan SL, Williams LJ, Pasco JA, et al. Relationship between diet and mental health in children and adolescents: a systematic review. Am J Public Health. 2014;104(10):e31-42. doi: 10.2105/AJPH.2014.302110 25208008 PMC4167107

[16] Soehner AM, Harvey AG. Prevalence and functional consequences of severe insomnia symptoms in mood and anxiety disorders: results from a nationally representative sample. Sleep. 2012;35(10):1367–75. doi: 10.5665/sleep.2116 23024435 PMC3443763

[17] Neckelmann D, Mykletun A, Dahl AA. Chronic insomnia as a risk factor for developing anxiety and depression. Sleep. 2007;30(7):873–80. doi: 10.1093/sleep/30.7.873 17682658 PMC1978360

[18] Ha T, Granger DA. Family relations, stress, and vulnerability: biobehavioral implications for prevention and practice. Fam Relat. 2016 Feb 22; 65(1):9–23.

[19] Ho YCL, Chew MSL, Ho CZH, Latib AB, Lee VSY, Lin GJ, et al. The validation of culturally appropriate scales to assess the family health climate in a multi-ethnic Asian population. Front Public Health. 2022 Oct 6;10. 10.3389/fpubh.2022.988525 PMC9584639 36276392

[20] Ho YCL, Chew MSL, Mahirah D, Thumboo J. Family resilience and psychological responses to COVID-19: a study of concordance and dyadic effects in Singapore households. Front Psychol. 2022 Mar 1;13. 10.3389/fpsyg.2022.770927 PMC8923423 35300159

[21] Preacher KJ, Hayes AF. Asymptotic and resampling strategies for assessing and comparing indirect effects in multiple mediator models. Behav Res Methods. 2008;40(3):879–91. doi: 10.3758/brm.40.3.879 18697684

[22] Fritz M, MacKinnon D. Required sample size to detect the mediated effect. Psychol Sci. 2007 Mar 4;18(3):233–9. 17444920 10.1111/j.1467-9280.2007.01882.x PMC2843527

[23] Walsh F. Strengthening family resilience. 2nd ed. New York, NY, US: The Guilford Press; 2006. xvi, 384–xvi, 384.

[24] Sixbey MT. Development of the family resilience assessment scale to identify family resilience constructs. University of Florida; 2005.

[25] Chew J, Haase AM. Psychometric properties of the family resilience assessment scale: a Singaporean perspective. Epilepsy Behav. 2016;61:112–9. doi: 10.1016/j.yebeh.2016.05.015 27337164

[26] Niermann C, Krapf F, Renner B, Reiner M, Woll A. Family health climate scale (FHC-scale): development and validation. Int J Behav Nutr Phys Act. 2014;11(1):30. doi: 10.1186/1479-5868-11-30 24593840 PMC4015295

[27] Kroenke K, Wu J, Yu Z, Bair MJ, Kean J, Stump T, et al. Patient health questionnaire anxiety and depression scale: initial validation in three clinical trials. Psychosom Med. 2016;78(6):716–27. doi: 10.1097/PSY.0000000000000322 27187854 PMC4927366

[28] Kroenke K, Spitzer RL, Williams JB. The PHQ-9: validity of a brief depression severity measure. J Gen Intern Med. 2001;16(9):606–13. doi: 10.1046/j.1525-1497.2001.016009606.x 11556941 PMC1495268

[29] Spitzer RL, Kroenke K, Williams JBW, Löwe B. A brief measure for assessing generalized anxiety disorder. Arch Intern Med. 2006 May 22;166(10):1092. 16717171 10.1001/archinte.166.10.1092

[30] Craig CL, Marshall AL, Sjöström M, Bauman AE, Booth ML, Ainsworth BE, et al. International physical activity questionnaire: 12-country reliability and validity. Med Sci Sports Exerc. 2003;35(8):1381–95. doi: 10.1249/01.MSS.0000078924.61453.FB 12900694

[31] Whitton C, Ho JCY, Rebello SA, van Dam RM. Relative validity and reproducibility of dietary quality scores from a short diet screener in a multi-ethnic Asian population. Public Health Nutr. 2018 Oct 7;21(15):2735–43. 30081973 10.1017/S1368980018001830 PMC10260988

[32] Fung TT, Hu FB, Wu K, Chiuve SE, Fuchs CS, Giovannucci E. The mediterranean and dietary approaches to stop hypertension (DASH) diets and colorectal cancer. Am J Clin Nutr. 2010 Dec 1;92(6):1429–35. 21097651 10.3945/ajcn.2010.29242 PMC2980967

[33] Buysse DJ, Reynolds CF 3rd, Monk TH, Berman SR, Kupfer DJ. The Pittsburgh Sleep Quality Index: a new instrument for psychiatric practice and research. Psychiatry Res. 1989;28(2):193–213. doi: 10.1016/0165-1781(89)90047-4 2748771

[34] Kline RB. Principles and practice of structural equation modeling. 4th ed. New York: Guilford Publications; 2016.

[35] Hayes AF. Introduction to mediation, moderation, and conditional process analysis. 2nd ed. New York: The Guilford Press; 2018.

[36] Hu L, Bentler PM. Cutoff criteria for fit indexes in covariance structure analysis: conventional criteria versus new alternatives. Struct Equa Modeling. 1999;6(1):1–55. doi: 10.1080/10705519909540118

[37] Singapore Department of Statistics. Population Trends 2020 [Internet]. Singapore; 2020. Available from: https://www.singstat.gov.sg/-/media/files/publications/population/population2020.pdf

[38] Singstat. Key Household Income Trends, 2020 [Internet]. Singapore; 2020. Available from: https://www.singstat.gov.sg/-/media/files/publications/households/pp-s27.pdf

[39] Chang L-Y, Wu C-C, Yen L-L, Chang H-Y. The effects of family dysfunction trajectories during childhood and early adolescence on sleep quality during late adolescence: resilience as a mediator. Soc Sci Med. 2019;222:162–70. doi: 10.1016/j.socscimed.2019.01.010 30641286

[40] Liu P-P, Yin P, Zhu Y-H, Zhang S, Sheng G-M. The correlation of family resilience with sleep quality and depression of parents of children with epilepsy. J Pediatr Nurs. 2021;56:e49–54. doi: 10.1016/j.pedn.2020.07.016 32800618

[41] Saltzman WR, Lester P, Beardslee WR, Layne CM, Woodward K, Nash WP. Mechanisms of risk and resilience in military families: theoretical and empirical basis of a family-focused resilience enhancement program. Clin Child Fam Psychol Rev. 2011 Sep 8; 14(3):213–30. 21655938 10.1007/s10567-011-0096-1 PMC3162635

[42] Porhcisaliyan VD, Wang Y, Tan NC, Jafar TH. Socioeconomic status and ethnic variation associated with type 2 diabetes mellitus in patients with uncontrolled hypertension in Singapore. BMJ Open Diabetes Res Care. 2021;9(1):e002064. doi: 10.1136/bmjdrc-2020-002064 34301679 PMC8728350

[43] Sigman-Grant M, Hayes J, VanBrackle A, Fiese B. Family resiliency: a neglected perspective in addressing obesity in young children. Child Obes. 2015;11(6):664–73. doi: 10.1089/chi.2014.0107 26447935

[44] Speirs KE, Hayes JT, Musaad S, VanBrackle A, Sigman-Grant M, All 4 Kids Obesity Resiliency Research Team. Is family sense of coherence a protective factor against the obesogenic environment?. Appetite. 2016;99:268–76. doi: 10.1016/j.appet.2016.01.025 26796029

[45] Williams LK, Veitch J, Ball K. What helps children eat well? A qualitative exploration of resilience among disadvantaged families. Health Educ Res. 2011 Apr 1;26(2):296–307. 21350037 10.1093/her/cyr004

[46] Badura P, Madarasova Geckova A, Sigmundova D, Sigmund E, van Dijk JP, Reijneveld SA. Do family environment factors play a role in adolescents’ involvement in organized activities? J Adolesc. 2017 Aug 2;59(1):59–66. 28582651 10.1016/j.adolescence.2017.05.017

[47] Black AP, D’Onise K, McDermott R, Vally H, O’Dea K. How effective are family-based and institutional nutrition interventions in improving children’s diet and health? A systematic review. BMC Public Health. 2017;17(1):818. doi: 10.1186/s12889-017-4795-5 29041899 PMC5645887

[48] Chase-Lansdale L, Brooks-Gunn J. Two-generation programs in the twenty-first century. Future Child. 2014;24(1):13–39. doi: 10.1353/foc.2014.0003 25518701

[49] Foltz JL, May AL, Belay B, Nihiser AJ, Dooyema CA, Blanck HM. Population-level intervention strategies and examples for obesity prevention in children. Annu Rev Nutr. 2012;32:391–415. doi: 10.1146/annurev-nutr-071811-150646 22540254 PMC10880737

[50] Hutchinson AD, Wilson C. Improving nutrition and physical activity in the workplace: a meta-analysis of intervention studies. Health Promot Int. 2012;27(2):238–49. doi: 10.1093/heapro/dar035 21733915

[51] Santos F, Sousa H, Gouveia ÉR, Lopes H, Peralta M, Martins J, et al. School-based family-oriented health interventions to promote physical activity in children and adolescents: a systematic review. Am J Health Promotion. 2023 Feb 22;37(2):243–262. 10.1177/08901171221113836 PMC9850376 36413351

[52] Gayatri M, Irawaty DK. Family resilience during COVID-19 pandemic: a literature review. Family J. 2022 Apr 14;30(2):132–8. 10.1177/10664807211023875 PMC8980846 35399750

[53] He Y, Li XS, Zhao J, An Y. Family resilience, media exposure, and children’s mental health in China during COVID-19. Family J. 2022 Oct 6;30(4):579–88.

[54] McDermott BM, Cobham VE, Berry H, Stallman HM. Vulnerability factors for disaster-induced child post-traumatic stress disorder: the case for low family resilience and previous mental illness. Aust N Z J Psychiatry. 2010;44(4):384–9. doi: 10.3109/00048670903489916 20307172

[55] Tang CS-K, Siu TSU, Chow TS, Kwok HS-H. The role of family resilience and pandemic burnout on mental health: a two-wave study in China. Int J Environ Res Public Health. 2023;20(5):3803. doi: 10.3390/ijerph20053803 36900809 PMC10001644

[56] Chang L, Chiang T. Family environment characteristics and sleep duration in children: maternal mental health as a mediator. Soc Sci Med. 2022 Dec;314:115450. 36257089 10.1016/j.socscimed.2022.115450

[57] Arora T, Grey I, Östlundh L, Alamoodi A, Omar OM, Hubert Lam K-B, et al. A systematic review and meta-analysis to assess the relationship between sleep duration/quality, mental toughness and resilience amongst healthy individuals. Sleep Med Rev. 2022;62:101593. doi: 10.1016/j.smrv.2022.101593 35462348

[58] Arden-Close E, McGrath N. Health behaviour change interventions for couples: a systematic review. Br J Health Psychol. 2017;22(2):215–37. doi: 10.1111/bjhp.12227 28150410 PMC5408388

[59] Dong F, Howard AG, Herring AH, Thompson AL, Adair LS, Popkin BM, et al. Parent-child associations for changes in diet, screen time, and physical activity across two decades in modernizing China: China Health and Nutrition Survey 1991-2009. Int J Behav Nutr Phys Act. 2016;13(1):118. doi: 10.1186/s12966-016-0445-z 27835960 PMC5106797

[60] Park SH, Cormier E. Influence of siblings on child health behaviors and obesity: a systematic review. J Child Fam Stud. 2018;27(7):2069–81. doi: 10.1007/s10826-018-1049-9

[61] Varma P, Conduit R, Junge M, Lee VV, Jackson ML. A systematic review of sleep associations in parents and children. J Child Fam Stud. 2021;30(9):2276–88. doi: 10.1007/s10826-021-02002-5

[62] Preacher KJ, Kelley K. Effect size measures for mediation models: quantitative strategies for communicating indirect effects. Psychol Methods. 2011;16(2):93–115. doi: 10.1037/a0022658 21500915

[63] Raudsepp L, Viira R. Influence of parents’ and siblings’ physical activity on activity levels of adolescents. Eur J Physical Educ. 2000;5(2):169–78. doi: 10.1080/1740898000050205

[64] Firth J, Solmi M, Wootton RE, Vancampfort D, Schuch FB, Hoare E, et al. A meta-review of “lifestyle psychiatry”: the role of exercise, smoking, diet and sleep in the prevention and treatment of mental disorders. World Psychiatry. 2020;19(3):360–80. doi: 10.1002/wps.20773 32931092 PMC7491615

[65] Saltzman WR, Lester P, Milburn N, Woodward K, Stein J. Pathways of risk and resilience: impact of a family resilience program on active‐duty military parents. Fam Process. 2016 Dec 6;55(4):633–46. 27597440 10.1111/famp.12238

[66] Saltzman WR, Pynoos RS, Lester P, Layne CM, Beardslee WR. Enhancing family resilience through family narrative co-construction. Clin Child Fam Psychol Rev. 2013 Sep 25;16(3):294–310. 23797387 10.1007/s10567-013-0142-2

[67] Lester P, Stein JA, Saltzman W, Woodward K, MacDermid SW, Milburn N, et al. Psychological health of military children: longitudinal evaluation of a family-centered prevention program to enhance family resilience. Mil Med. 2013;178(8):838–45. doi: 10.7205/MILMED-D-12-00502 23929043 PMC4020707

[68] Lester P, Liang L-J, Milburn N, Mogil C, Woodward K, Nash W, et al. Evaluation of a family-centered preventive intervention for military families: parent and child longitudinal outcomes. J Am Acad Child Adolesc Psychiatry. 2016;55(1):14–24. doi: 10.1016/j.jaac.2015.10.009 26703905

[69] Saltzman WR The FOCUS family resilience program: an innovative family intervention for trauma and loss. Fam Process. 2016 Dec 13;55:(4):647–59. 27734461 10.1111/famp.12250

