# Supplementary material for: Family resilience influences on individual physical activity, diet and sleep quality: Family health climate and biobehavioural reactivity as driving mediators
Source: PLoS One. 2025 May 5;20(5):e0322612. doi: 10.1371/journal.pone.0322612 (PMC12052107; doi:10.1371/journal.pone.0322612)
Supplement: S1 Appendix — This appendix contains the summary results of the inversed parallel mediation models in comparison with the study findings. (DOCX) [file pone.0322612.s001.docx]

**S1. Appendix.**

Summary results of inversed parallel mediation models with study findings

Table A1 presents the mediating effects of biobehavioural reactivity and family health climate on respective lifestyle behaviours as they relate to family resilience. The overall model fit indices for the inversed parallel mediation models are shown in Table A2.

The inversed models demonstrated mediation effects like those of the original models, except for sleep quality. Notably, these inversed models exhibited a poorer overall fit than the original hypothesized models.

Both model types indicate bidirectional mediation effects of family health climate on diet quality and family resilience, as well as biobehavioural reactivity on sleep quality and family resilience. A significant new unidirectional mediation pathway was identified: family health climate on individual sleep quality affecting family resilience (β = -.069, *p* = .01), which contrasts with the non-significant indirect effect observed in the original model (*p* > .05).

Table A1. Mediation effects of PHQ-ADS and FHC with family resilience.

| Model | Path | β | SE B | *p*-value |
| --- | --- | --- | --- | --- |
| Individual physical activity to family resilience  (X1 🡪 M1\|M2 🡪 Y) | Total effect (c) | .17 | .0057 | **< .001** |
|  | Direct effect (c’) | .021 | .0051 | .27 |
|  | Total indirect effect (a x b) | .15 | . 0037 | **< .001** |
| (X1 🡪 M1 🡪 Y) | M1 indirect effect | .018 | .0015 | .56 |
| (X1 🡪 M2 🡪 Y) | M2 indirect effect | .15 | .0037 | **< .001** |
| Individual diet quality to family resilience  (X2 🡪 M1\|M2 🡪 Y) | Total effect (c) | .19 | .16 | **< .001** |
|  | Direct effect (c’) | .057 | .17 | .18 |
|  | Total indirect effect (a x b) | .14 | .13 | **< .001** |
| (X2 🡪 M1 🡪 Y) | M1 indirect effect | .019 | .054 | .12 |
| (X2 🡪 M2 🡪 Y) | M2 indirect effect | .13 | .13 | **< .001** |
| Individual sleep quality to family resilience  (X3 🡪 M1\|M2 🡪 Y) | Total effect (c) | -.16 | .27 | **.0010** |
|  | Direct effect (c’) | -.015 | .29 | .79 |
|  | Total indirect effect (a x b) | -.15 | .20 | **< .001** |
| (X3 🡪 M1 🡪 Y) | M1 indirect effect | -.13 | .18 | **< .001** |
| (X3 🡪 M2 🡪 Y) | M2 indirect effect | -.069 | .15 | **.011** |

Notes. X = lifestyle behaviours; M1 = PHQ-ADS; M2 = FHC; Y = family resilience; β = standardised regression coefficient; SE B = bootstrap standard error; M1|M2 = M1 and M2 as parallel mediators.

Table A2. Overall model fit indices for inversed parallel mediation models.

| Model | χ^2^/df | CFI | TLI | RMSEA | SRMR | Overall model fit |
| --- | --- | --- | --- | --- | --- | --- |
| Individual physical activity to family resilience  (X1 🡪 M1\|M2 🡪 Y) | 40.58/25 | 0.949 | 0.889 | 0.040  (*p* > .05) | 0.029 | Fair |
| Individual diet quality to family resilience  (X2 🡪 M1\|M2 🡪 Y) | 42.87/25 | 0.941 | 0.874 | 0.042  (*p* > .05) | 0.030 | Fair |
| Individual sleep quality to family resilience  (X3 🡪 M1\|M2 🡪 Y) | 49.83/25 | 0.944 | 0.880 | 0.050  (*p* > .05) | - | Fair |

Notes. X = lifestyle behaviours; M1 = PHQ-ADS; M2 = FHC; Y = family resilience; M1|M2 = M1 and M2 as parallel mediators.
